# Feature Adaptive Sampling for Scanning Electron Microscopy

**DOI:** 10.1038/srep25350

**Published:** 2016-05-06

**Authors:** Tim Dahmen, Michael Engstler, Christoph Pauly, Patrick Trampert, Niels de Jonge, Frank Mücklich, Philipp Slusallek

**Affiliations:** 1 German Research Center for Artificial Intelligence GmbH (DFKI), 66123 Saarbrücken, Germany; 2 Saarland University, 66123 Saarbrücken, Germany; 3 INM-Leibniz Institute for New Materials, 66123 Saarbrücken, Germany

## Abstract

A new method for the image acquisition in scanning electron microscopy (SEM) was introduced. The method used adaptively increased pixel-dwell times to improve the signal-to-noise ratio (SNR) in areas of high detail. In areas of low detail, the electron dose was reduced on a per pixel basis, and a-posteriori image processing techniques were applied to remove the resulting noise. The technique was realized by scanning the sample twice. The first, quick scan used small pixel-dwell times to generate a first, noisy image using a low electron dose. This image was analyzed automatically, and a software algorithm generated a sparse pattern of regions of the image that require additional sampling. A second scan generated a sparse image of only these regions, but using a highly increased electron dose. By applying a selective low-pass filter and combining both datasets, a single image was generated. The resulting image exhibited a factor of ≈3 better SNR than an image acquired with uniform sampling on a Cartesian grid and the same total acquisition time. This result implies that the required electron dose (or acquisition time) for the adaptive scanning method is a factor of ten lower than for uniform scanning.

SEM is a widely used microscopy technique with applications in fields such as material science, life science, and semiconductor research. The specimen is scanned by a focused electron beam, and back scattered electrons or secondary electrons are detected with the respective detectors. SEM techniques can be extended to three-dimensional (3D) imaging using several approaches^1^. In focused ion beam scanning electron microscopy (FIB/SEM), a specimen is imaged by sequentially removing thin layers of material using a focused ion beam. Each resulting surface is then scanned using a focused electron beam such that 3D information is acquired layer-by-layer^2^,^3^,^4^,^5^. A similar technique is STEM in which the image is acquired via scanning the electron beam but here transmitted electrons are used for contrast. In STEM tomography, the specimen is imaged at different tilt angles of the specimen. A number of recording schemes has been proposed including single axis tilt series^6^, double axis tilt series^7^,^8^, and conical tilt series^9^,^10^. Alternatively, 3D information is obtained via recording of a focal series^11^,^12^. Recently, the combined tilt- and focal series has been introduced as an additional option^13^,^14^. Common to all these recording schemes is the need to acquire a set of images that are used to reconstruct a 3D dataset.

Due to the large number of pixels in a dataset, the pixel-dwell time, which is the amount of time the electron beam illuminates a single pixel, is a crucial parameter for the aforementioned techniques. The pixel-dwell time required for a sufficiently high SNR leads to large data acquisition times, in particular for 3D techniques. For modern microscopy equipment operating with high brightness field emission electron sources, it is typically impossible to decrease the pixel-dwell time, since the probe current in the same spot size cannot be increased on account of the limited brightness of the electron source. As an example, consider the acquisition of a 3D dataset using FIB/SEM serial sectioning. The size of FIB/SEM tomography datasets is typically in the range of 1024^2^ ×  256 voxels^15^. With a typical dwell time of 30 *μs*, the total time for the image acquisition is in the range of 2 hours. If the data from FIB/SEM tomography is extended to the range of 2048^2^ ×  512 or even 4096^2^ ×  512 voxels, the total image acquisition time at 30 *μs* dwell time would be in the order of 18 hours to 3 days.

Another critical factor for most samples under investigation is radiation damage^16^,^17^. The effect is the strongest in organic samples such as polymers or biological specimen^18^,^19^. Beam induced damage in biological specimen expresses itself primarily in the form of sample shrinkage perpendicular to the sample plane^20^ and damage to the structure of the sample.

There are several standard strategies to reduce image acquisition time and/or electron dose with characteristic drawbacks. The electron microscope can be adjusted for a higher probe current resulting in a higher electron dose per time unit and, thus, a shorter acquisition time, but the spatial resolution will be reduced in most cases. Reducing the dwell time at a given probe current will lead to a lower SNR, and thus limits the information content of the image. Posterior filtering of the image, e.g. mean filtering, is frequently used to improve image quality after image acquisition, but the fine structures in the image are blurred and image sharpness is lost.

In order to circumvent the drawbacks of low SNR at high scan rates, more advanced scanning strategies have been developed. Oho *et al.*^21^ proposed a method called “aquincuncial”, i.e. non-Cartesian scanning in under-sampled conditions. During conversion from a non-Cartesian grid to a Cartesian grid, the number of pixels doubles with only a small reduction in image quality, at least in images with structures aligned in x- and y-direction. Timischl^22^ proposed a dynamic scanning method based on adjusting the scanning speed to the amount of detected electrons. In bright appearing sample areas, more electrons are detected, and the scanning speed can be increased. In this study, we propose to extend this idea by using adaptive scan rates, and use a criterion for the scan rate based not on the value of an individual pixel, but on an analysis of the entire image.

The algorithmic idea of image based adaptive sampling originates from the field of realistic image synthesis, for example in movie production. Algorithms in this field are typically based on the ray tracing^23^,^24^ method. Hereby, a realistic, two-dimensional image is synthetically generated from a 3D scene by computing the color value of each pixel. This is achieved by creating a ray starting from the pixel, and computationally simulate light propagation along the ray. As each pixel represents a small area, not a point, on the camera plane, one position inside the pixel is selected as the starting point for the ray, called the sample position. Typically, several samples are evaluated and averaged to achieve better image quality (“anti-aliasing”). However, the computational cost of the lighting simulation per ray is high in general, so it is desirable to reach the best possible result with as little samples as possible. Various adaptive sampling schemes have been proposed and will be described in the following.

In the method called adaptive super sampling^23^, rays are cast through the corners of a pixel. If the four values do not differ from each other by too much, their average is used for the pixel. If not, more rays are cast-from the center of the pixel and the centers of the pixel edges. This subdivision continues as needed until some preset limit on the number of subdivisions is reached. An alternative approach is adaptive super sampling using Poisson-disk sampling^25^, whereby samples are distributed randomly and some regions of the image may require extra sampling, to avoid aliasing. Similarly, a method proposed by Painter and Sloan^26^ also uses adaptive super sampling based on the image space, but their procedure starts from above the pixel level.

An incremental approach to adaptive sampling is taken in^27^, where a sequence of images is generated with incrementally improved quality. The first images are generated by casting a limited set of rays (less than one per pixel) and interpolating the results. Subsequent images are formed by casting additional rays and maximizing their impact by distributing them according to a measure of local image complexity.

Since those early contributions, adaptive sampling methods have a permanent place in computer graphics research and applications. However, the focus of research has shifted to advanced sampling techniques that make use of prior knowledge of the physical nature of light propagation and thus are not readily applicable to electron microscopy. In this study, we apply the principle idea of image based adaptive sampling to the image formation process of two dimensional scanning electron microscopy. However, the same method could in principle be applied to three dimensional FIB/SEM tomography, STEM tomography or even scanning x-ray tomography^28^.

The proposed method^29^ consists of an adaptive scanning method based on image analysis of an entire image. In order to achieve the seemingly paradox goal of scanning an image based on its own, a-priori unknown, characteristics, a two-step scanning is performed. An initial image is acquired on a conventional Cartesian grid. This initial scan is recorded with a small pixel-dwell time. This information is used to estimate image characteristics resulting in a mask that contains areas of interest, mainly the phase boundaries. A second scanning step is then performed to gather additional information. In this step, the high interest areas (HIA) are scanned again at a highly increased pixel-dwell time resulting in a sparse dataset of drastically improved SNR. The final image is then formed by filtering the initial dataset to remove noise. This is combined with the second dataset to bring in high frequency detail in the HIA.

## Material and Methods

### Samples

Cast iron samples used in this study were provided by Neue Halberg Guss GmbH, Saarbrücken, Germany. The microstructure consists of a pearlitic iron matrix with graphite inclusions. The carbon content is approximately 3.5 *wt*% and the silicon content is approximately 2 *wt*% for both samples. The first sample has nodular graphite morphology and was taken from a sand casting of a crankshaft for internal combustion engines. The second sample has lamellar graphite inclusions and was taken from a sand casting of a crankcase for internal combustion engines.

### Microscope

The microscope employed in this study was a FEI Helios NanoLab600 equipped with a Shottky field emission electron source. The electron beam was set to an accelerating voltage of 5 *kV* and a probe current of 340 *pA*. Images were acquired with the Everhart-Thornley secondary electron detector at a working distance of 4 *mm*.

### Software

**Figure 1:**
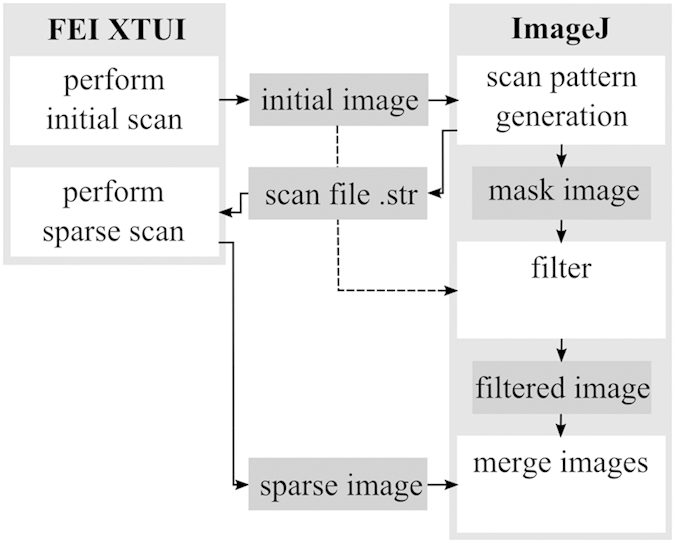
The process of image acquisition. An initial image was acquired at low pixel-dwell time in XTUi. The image was analyzed in ImageJ and two files (scan file and mask image) were generated, both representing the classification of the image in a high interest area (HIA) and a filter region. A second sparse image was then acquired for the HIA with much larger pixel-dwell time. The initial image was filtered using an adaptive filter kernel based on the mask image. The sparse image and a filtered version of the initial image were combined to form the final result.

The basic image acquisition performed in this study was executed using FEI XTUi 3.8.9. The software is equipped with basic beam guiding functions for electron and ion beam patterning. All functions specific to the methods are presented in this paper and described below were developed as Java Plugins to the ImageJ derivative FIJI^30^. The combination of the custom software and FEI XTUi resulted in a prototype workflow (Fig. 1) for the recording of images using the proposed adaptive sampling pattern.

### Image Acquisition

The method presented in this study started with the acquisition of a 1024^2^ pixel image at a low pixel-dwell time of 1 *μs* recorded with 16 bit per pixel. The image was transferred to ImageJ for further processing. It was analyzed automatically and classified in two regions. The first region (“filter region”) showed smooth gradients, i.e. only low frequency variation of the gray values. The second regions, the HIA, consisted of strong discontinuities of the gray values, i.e. it contained the pixels showing edges and other sharp features.

The classification was based on the idea of detecting maxima in gradient magnitude values of a Gaussian-smoothed version of the image. Details on the algorithm are given in^31^. We used the existing implementation of the algorithm in the software package FeatureJ. First, the image was converted to 32 bit gray scale. Next, the edge detection algorithm was executed using the parameters *“smoothing scale”* = *1.0* and *“suppress non-maximum gradients”* = *false*, which resulted in an intermediate image representation. Next, the image was converted to a binary (black and white) representation, i.e. thresholding was applied. This thresholding was performed in such a way that a fixed number of pixels was classified as belonging to the HIA. In order to determine the correct thresholding value to match this condition, a *256* bin histogram of the intermediate representation was computed. The running sum of the histogram was computed to find the approximate value that represented a certain percentage of pixels with highest intensity. Thresholding was then applied to the image according to this value and it was converted to black and white.

The size of the HIA was selected according to two criteria. Firstly, the resulting mask had to consist of closed shapes around all phase boundaries in the sample, which was required by the filtering procedure described below. Secondly, the size of the HIA should be minimal in order to reduce the average pixel dwell time. The value that fulfills those requirements depends on the sparsity of the gradient image, i.e. how “dense” the image appears visually and had to be entered by the user. This user interaction was supported by a real-time preview of the resulting mask. The question if an image consists of closed shapes or not can in principle also be solved algorithmically, so this step might be automated in the future.

Alternatively, a gradient magnitude value for the thresholding could be specified directly. In this case, the software would select a varying amount of pixels for the HIA to reach a constant quality level of the mask. This alternative approach might be beneficial in the case of 3D sectioning, if subsequent slices have a different structure from the first slice and thus the size of the HIA needs to be adapted per vertical image position.

As the operating software used in this study does not support acquiring pixel-selective images, we used features originally intended for ion beam structuring. A trace file in the format PIA Stream File Extension (.str) was generated to transfer the shape of the HIA to the software XTUi 3.8.9. Stream files directly access the digital 16 bit scan generator and are typically used for milling complex geometries with the ion beam. However, the stream file can also be used to control the electron beam. To use stream files for image acquisition, we enabled the built-in real time monitor (RTM). This feature activates the secondary electron detector while the stream file is running and can be used to acquire a pixel-selective image according to the desired HIA. This way, one can achieve fine granular control in the image acquisition process and generate images that have their pixels arranged in arbitrary patterns.

To acquire the desired sparse image, the stream file was loaded using the patterning interface of the software. By starting the “patterning” process with activated RTM, the beam was switched on and data was recorded only in regions specified by the coordinates in the stream file. Hereby, the feature “blackout pattern area for RTM” was used to erase remaining signals in the detector prior to recording. The acquired pixels were then recorded in the RTM and could be saved and used for further image processing outside the microscope software.

The trace file mentioned above was generated in such a way that the beam scanned the image row by row. The beam was turned on (“unblanked”) when entering the HIA. Inside the HIA, every pixel was scanned with a larger dwell time resulting in an improvement of SNR compared to the initial image. When leaving the HIA, the beam was turned off (“blanked”) and the microscopy skipped to parts of the image representing the filter region. This way, the software XTUi generated a second image, containing only pixels in the rescanning region, but at improved SNR. The pixels representing the filter region contained zeros.

### Filtering

**Figure 2:**
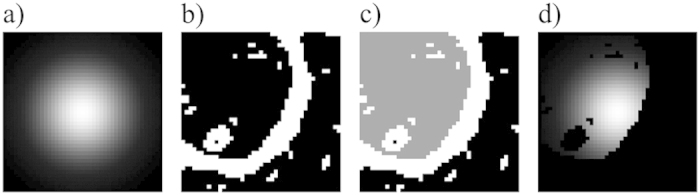
Structure of the filter kernel. The image was filtered using a different filter kernel for each pixel. (**a**) The filter was designed based on a discrete Gaussian as a starting point. (**b**) The Gaussian kernel was modified by considering the shape of the filter area around the pixel. The filter area is shown in black, the high interest area (HIA) in white. (**c**) A flood fill algorithm was used to detect only those pixels reachable from the center (gray). (**d**) The Gaussian was set to zero for all pixels not contained in the mask and re-normalized.

In the filter area of the image, a band-limiting image filter was applied to reduce the image noise. The filter was designed based on a Gaussian filter kernel because of the well understood band-limiting properties of this kernel. Filtering with a Gaussian filter worked without problems inside the filter area but resulted in artifacts at the border to the HIA in cases where the filter kernel overlapped the HIA. Therefore, the filter kernel was modified per pixel, i.e. a different filter kernel was used for each pixel. The kernel was generated by first computing a discrete representation of a 2D Gaussian kernel of width σ (Fig. 2a). Pixels outside a cutoff of 3σ were assumed to be zero. The filter was then multiplied pixel per pixel with a mask, i.e. the filter value was set to zero for all pixels outside this mask. As a mask, the shape of the HIA around the filtered pixel was used (Fig. 2b). This avoided that the filter used pixels from the HIA to compute a pixel value in the filter area. However, multiplication with the mask was insufficient. For larger Gaussian kernels, the filter kernel sometimes overlapped narrow strips of the HIA and reached a second patch of the filter area. This second patch typically belonged to a different phase of the sample and should not be considered either. A flood-fill algorithm was therefore used starting at the center of the kernel. All pixels not reachable by the flood-fill were also excluded from the mask (Fig. 2c). Finally, the kernel had to be re-normalized to ensure that pixel values sum up to one and thus the resulting filter preserves intensity (Fig. 2d).

The filtering method is an edge preserving smoothing operator, such as the well-known bilateral filter^32^ or anisotropic diffusion^33^. However, our method uses an explicit representation of the features to be preserved, which are known a-priori from the previous step in the method gives as mask image. In this regard, the filter is more comparable to a guided filter^34^, albeit avoiding the complicated optimization steps.

### Adapation of noise level

Using the filter described above and choosing a Gaussian with sufficiently large value of σ , the noise in the filter area could be removed to almost arbitrary low levels. In the HIA, the noise level is reduced by scanning the pixels again at increased pixel-dwell time, respectively electron dose. The reduction of noise in the HIA is not arbitrary, though, but depends on the ratio of dwell times of the initial scan and the second scan. Typical values are 300 *ns* for the first scan and 30 *μs* for the second scan, such that pixel-dwell times that are 100 *x* higher resulting in a SNR improvement of a factor of 10, which still involves clearly visible noise. When combining filter area and HIA to form the final image, different parts of the image have different noise levels. This can easily be avoided by mixing the filtered version of the HIA with the unfiltered version in the right ratio.

## Results

### Benchmarking and quantitative assessment

**Figure 3:**
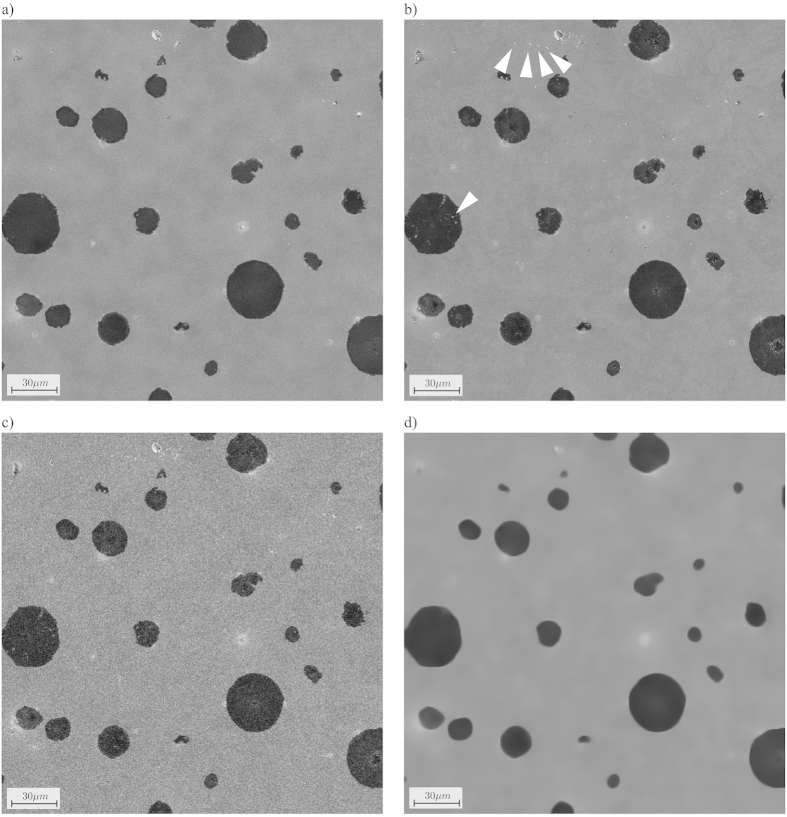
Overall performance of the adaptive sampling method. (**a**) Image recorded with our method using an average pixel-dwell time of 3 *μ s* (initial scan 300 *ns*, sparse scan 27 *μ s* for 10% of pixels). (**b**) Image recorded with a uniform pixel-dwell time of 60 *μ s* as ground truth. The image shows some small details that are missing in the sparse scan (examples are indicated by white arrows). (**c**) Image recorded with uniform sampling at the same pixel-dwell time (3 *μ s* per pixel). (**d**) Image recorded at 3 *μ s* per pixel and filtered using an anisotropic diffusion operator.

In order to benchmark the proposed method, several images of cast iron with nodular graphite inclusions were recorded. The investigated image consisted of a signal and deviations, which were composed of noise and systematic errors. For the conditions used in this study, the noise was dominated by statistical noise that is a consequence of the limited number of electrons per pixel. Systematic deviations included among others (1) microscope drift, which can occur mainly at high magnifications or unstable operation conditions, (2) a convolution with the point spread function (PSF), and (3) a deformation of the PSF that is a consequence of non-intermediate signal response of the scanning unit and mainly occurs at very high scan rates. All systematic errors were negligible compared to the noise and thus were ignored for this study. The image was recorded using an initial scan with 300 *ns* pixel-dwell time and a sparse scan using 27 *μs* for an HIA of 10%, which results in an average pixel-dwell time of 3 *μs* (Fig. 3a). A ground truth scan was performed using a high pixel-dwell time of 60 *μs* (Fig. 3b). As comparison, a conventional scan of the same sample was performed using uniform scanning with a pixel-dwell time of 3 *μs* (Fig. 3c). As image processing operations might also be applied to images recorded using uniform scanning, a state-of-the-art edge preserving smoothing filter was applied. We have choosen an anisotropic diffusion operator^33^ with the following settings: number of iterations: 200, smoothings per iteration: 1, diffusion limiter along minimal variations: 0.2, diffusion limiter along maximal variations: 0.9, time step: 2, edge threshold height: 5.0.

As can be seen by comparing Fig. 3a–c, the image recorded using the adaptive sampling technique looks smoother and contains less image noise. By comparing Fig. 3a,b, one of the drawbacks of the adaptive sampling method can be seen, namely the fact that some smaller details are missing entirely in the sparse scan. Examples are marked with white arrows in Fig. 3b. An interesting benchmark is to compare the image acquired using adaptive sampling (Fig. 3a) to the result obtained by combining uniform sampling at the same average pixel-dwell time and applying state-of-the-art a-posteriori image processing methods (Fig. 3d). As can be seen, the the anisotropic diffusion filter is effective at removing noise, but also creates a slighly blurred image that misses even more details compared to the adaptive sampling scheme.

By computing the pixel error, i.e. the per-pixel difference between the ground truth image and the images recorded with different settings, signal and deviations could be separated. We found that systematic errors can be neglected in our case and in the following, we assume that deviations consist of noise only. We thus considered three values for each pixel: the value in the ground truth image (signal only), the value in the image recorded with the respective method (signal+ noise) and the difference (noise only). This allowed us to quantify the impact of the adaptive sampling and compute the SNR according to the formula mean of signal divided by standard deviation of noise (Table 1). Additionally, we computed peak signal-to-noise-ratio (PSNR)^35^ and structural similarity index (SSIM)^36^. In the following, all error values are given as difference between pixel value in the respective scan and the same pixel value in the ground truth image. All values are expressed as percentage of the maximum pixel intensity of the reference scan for comparability. The adaptive sampling scheme with adaptation of noise level produces an average pixel error of 1.88% ±  1.65%. This is significantly lower than the uniform scan, which has a pixel error of 4.42% ±  3.46%. Another observation is that the standard deviation of the pixel error is similar to the average value, which can be expected for an error that is dominated by noise. The uniform scan filtered with the isotropic diffusion filter has a pixel error of 1.58% ±  1.81%, which is even lower than the adaptive sampling. However, one has to keep in mind that a large part of the pixel error in the case of adaptive sampling is a result of the adaptation of the noise level (see methods section). If the noise level adaptation is turned off, the pixel error for the adaptive sampling is reduced to 1.30% ±  1.35%, which is significantly better than the values measured for the images acquired with uniform sampling and isotropic diffusion filtering. When considering the maximum pixel error, the adaptive sampling scheme produces a better result than the uniform scheme with and without noise level adaptation and with and without filtering.

### Evaluation of the mask generation procedure

The mask generation procedure was applied with different settings on a sample of cast iron with nodular graphite inclusions. A HIA that contains 5% of all pixels turned out to be sufficient as representation of gradients since it contained all phase boundaries. Allowing for more than 5% led to a highly segmented HIA and did not provide additional benefits. However, the sample used was favorable for this filtering method, so an HIA size of more than 5% might be required for samples with a denser gradient, such as biological specimen or material science samples with a more complex structure.

The same experiment was conducted on a specimen consisting of cast iron with lamella graphite inclusions. The sample had comparable characteristics as the nodular graphite sample in the sense that there were two clearly separated phases. However, the lamella structure of the graphite inclusions was more complex, which constituted a more difficult case for the edge detection algorithm. The edge detection algorithm with a HIA size of 10% of the total image size gave optimal results in the sense that all phase boundaries were included.

### Influence of noise in initial image

Next, the influence of noise in the initial image on the mask generation was investigated. Images were acquired from the lamella graphite sample with different pixel-dwell times of 100 *ns*, 300 *ns*, 1 *μs*, and 3 *μs*. We found that for these different pixel-dwell times, i.e. for different SNR levels in the initial image, the mask generation algorithm gave optimal results with different input settings (Table 2). The criterion for an acceptable mask was that all particles were surrounded by closed shapes, i.e. all phase boundaries were visible on the mask without gaps. The main parameters were the size of the Gaussian filter for the edge detection algorithm σ (in pixels) and the size of the high interest area A, normalized to the total size of the image (in percent).

**Figure 4:**
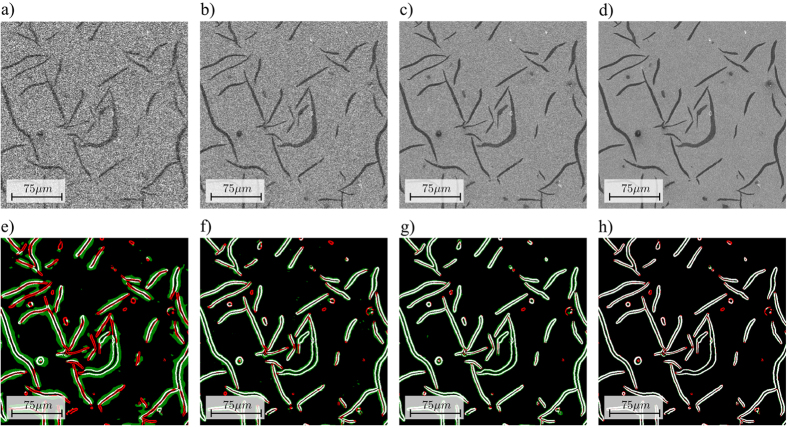
Impact of the pixel-dwell time of the initial image on the shape of the high interest area (HIA). The top row shows the acquired images at (**a**) 100 *ns*, (**b**) 300 *ns*, (**c**) 1 *μ s*, and (**d**) 3 *μ s*. The bottom row shows the generated HIA in white. The differences to a ground truth HIA generated from an image acquired with 60 μ s pixel-dwell time are color coded. Red pixels are missing in the HIA, i.e. might lead to image errors. Green pixels are additional pixels in the HIA, i.e. will waste pixel-dwell time during the sparse scan. Results are shown for (**e**) 100 *ns*, (**f**) 300 *ns*, (**g**) 1 *μ s*, and (**h**) 3 *μ s*.

As can be seen, a lower SNR in the initial image required an increased HIA to avoid missing edges, because the determination of the optimal shape was less accurate. Even with an increased HIA size of 20%, we found that a pixel-dwell time of 100 *ns* or less gives unsatisfactory results (Fig. 4e). For a pixel-dwell time of 300 *ns*–1 *μs*, the results were acceptable (Fig. 4f,g). An image acquired with a pixel-dwell time of 3 *μs* additionally allowed reducing the HIA size from 15% to 10% of the total image size (Fig. 4h).

The required electron dose for the initial scan also depended on the contrast of the image. For a sample with several phases, the SNR of the image can be defined as the difference of the gray values of the two phases with the most similar gray values, divided by the standard deviation of the noise in the image. While the error-free detection of every pixel in the edge would require an SNR of 4–5 according to the Rose criterion^37^, we found experimentally that an SNR of 1.4 to 2.3 gives satisfactory results with the used edge detection algorithm (see also Table 2). The difference to Rose can be explained by the fact that (1) the edge detection does detect larger structures, not individual pixels, and (2) a certain amount of false positives, i.e. pixels that are accidentally classified as belonging to the HIA are acceptable. From this observation, a rule of thumb can be derived to choose the appropriate pixel dwell time for the initial scan, assuming the basic structure of the sample is known. The pixel dwell time should be selected such that the SNR according to the definition above is between 1.4 for a sample with coarser structures and 2.3 for a sample with finer structures.

### Influence of noise level adaptation

**Figure 5:**
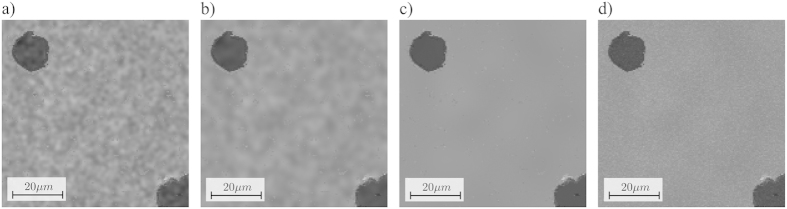
Results of the noise adaptation procedure. (**a**) A filter kernel based on a Gaussian with σ  =  1.5 pixels resulted in visually unpleasant artifacts in the medium frequency range. (**b**) A Gaussian with σ  =  2.5 pixels showed comparable artifacts, albeit at lower frequencies. (**c**) A filter based on a sufficiently large Gaussian with σ  =  10 pixels removed those artifacts. However, the filter region now had a much lower noise level than the high interest area (HIA), which also was clearly visible. (**d**) By mixing the filtered version of the signal from the initial scan with the unfiltered version in the right ratio, the noise level could be adapted and the filter region could no longer be detected easily.

As explained in the methods section, a critical factor were the band limiting characteristics of the used smoothing filter. Choosing a moderate size of the Gaussian filter (about 1< σ < 5 pixels) resulted in clearly visible dominant structures with a spatial frequency about the same distance (Fig. 5a,b). Even worse, the structures did not stretch over the full extent of the image, they only covered the filter area. As a consequence, pixels of the HIA that do not contain these artifacts appeared visually separated and were most likely perceived as artifacts as well. In order to avoid these problems, the filter kernel had to be chosen sufficiently large (σ  ≫  5 pixels). In this case, however, the noise level in the filter area was much lower than the noise level in the HIA, so the two areas of the image still did not merge visually, and the HIA remained clearly visible to the human eye (Fig. 5c).

The solution to this situation was to adapt the noise levels in the filter area and the HIA. This could be achieved by replacing the pixel values in the filter area with a linear combination between the initial signal and the filtered signal according to the formula:


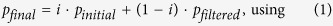



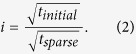


Hereby, *p*_*final*_ is the final pixel value, *p*_*filtered*_ the filtered pixel value, and *p*_*initial*_ is the unfiltered pixel value. The factor *i* was computed as the ratio of the square root of the pixel-dwell time of the initial scan *t*_*initial*_ and the pixel-dwell time of the sparse scan *t*_*sparse*_. The formula can be motivated using a statistical argument. A random variable estimated by *N* binary samples has a standard deviation proportional to *1/N*^*1/2*^. In the context of SEM gray values, this means that a pixel gray value suffers from statistical noise proportional to the inverse of the square root of the pixel-dwell time, assuming constant beam current. In the case of a sufficiently large filter kernel, it can furthermore be assumed that there is no visible noise in the filtered version of the signal. This means that the factor *i* is the ratio of the noise level in the initial and the sparse image. Thus, combining the noise-free signal *p*_*filtered*_ and the noisy signal *p*_*initial*_ in this ratio results in the desired amount of statistical noise in the final pixel value (Fig. 5d).

### Scalability to higher magnifications

**Figure 6:**
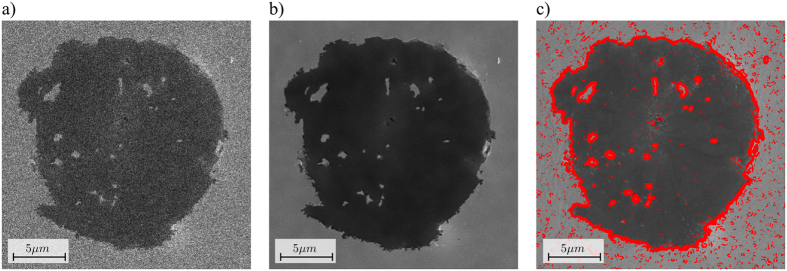
Results at higher magnifications. (**a**) Image recorded with a pixel size of 35 nm and a pixel-dwell time of 300 *ns*. (**b**) Result of the adaptive sampling with an initial scan of 300 *ns* and a sparse scan with A =  0.1 and 30 *μ s* pixel-dwell time. (**c**) Ground truth image scanned with a dense scan of 60 *μ s* pixel dwell time, with the high interest area (HIA) indicated in red.

In order to test scalability of the method to higher magnifications, images of the nodular graphite cast iron sample were recorded using a pixel size of 35 *nm*. The initial scan was performed with a pixel-dwell time of 300 *ns* (Fig. 6a). The mask was then generated with a HIA area of *A* =  10%. The sparse scan was performed with a pixel-dwell time of 30 *μs*. The filtering was performed with a filter kernel based on a Gaussian with σ  =  10 pixels and the images were combined to a final image with adapted noise level (Fig. 6b). At this high magnification, even a small drift that occurs between the initial scan and the sparse scan may lead to visible artifacts in images. However, in our tests we did not encounter microscope drift.

## Discussion

We propose a new method to reduce the average pixel dwell time in SEM by an adaptive distribution of electron radiation. Parts of the sample that show high frequency details are exposed to longer pixel-dwell times while smooth parts of the image are scanned more quickly and the resulting noise is removed in software using image processing filters. In order to understand the amount of pixel-dwell time that can be gained by the method, one must consider three parameters: the pixel-dwell time *t*_*initial*_ for the initial image, the size of the high interest area *A* normalized by the total size of the image, and the pixel-dwell time for the sparse image *t*_*sparse*_. The average pixel-dwell time is then given by *t*_*avg*_ = *t*_*initial*_ + *A · t*_*sparse*_. Typical values are *t*_*initial*_ =  300 *ns*, and scanning of 10% of the area (*A* = *0.1*) using *t*_*sparse*_ = 27 *μs.* In this case, *t*_*avg*_ = *3* *μs* and the total acquisition time adds up to *t*_*avg*_ multiplied by the number of pixels. According to our measurements, the image based adaptive sampling method improved the SNR by a factor of ≈ 3 compared to a uniform sampling at the same average pixel-dwell time. This implies that the electron dose can be reduced by a factor of ten for adaptive scanning compared to uniform sampling, since the SNR scales with the square root of the amount of electrons.

Advantages of the method are two-fold. For the acquisition of a high resolution 3D dataset with FIB/SEM, the total acquisition time is one of the main limiting factors of the technique. Particularly for the acquisition of high-resolution 3D datasets, pixel-dwell times can dominate the total beam time of days or weeks and, thus, limit the feasibility of data acquisition. The proposed method allows to reduce acquisition times by a factor of ten depending on the sample.

Saving beam time is not the only motivation for reducing pixel-dwell times. Many samples, like biological samples, polymers, and ceramics, are sensitive to electron dose. For those cases, imaging means finding a compromise between beam-induced radiation damage and noise. The method proposed in this paper can reduce the average pixel-dwell time and, thus, helps to avoid radiation damage without introducing noise. However, the total electron dose will no longer be distributed evenly over the entire specimen, but focused at phase boundaries. While a lot of research exists on the effects of radiation damage on different kind of specimen^18^,^19^,^38^,^39^,^40^, none of this research considers inhomogeneous spatial distribution of radiation. The usefulness of the adaptive scanning scheme for the imaging of dose sensitive specimen will largely depend on the question how different kinds of specimen behave under locally focused radiation.

Formation of beam damage is a complex process, the predominant effects are typically (1) ionization, (2) knock-on damage, i.e. removing individual atoms from the matrix, both followed by structural collapse in the form of sample shrinkage and (3) sample heating^41^. For both, ionization and knock-on damage, we expect the effects to be highly localized, i.e. to occur mainly at the phase boundaries where the highest electron dose is placed. The most prominent radiation damage effect with conventional methods is sample shrinkage. This type of damage is expected to be reduced or even avoided altogether using the non-uniform scanning because small structures exposed to high dose will be embedded in a stabilizing matrix of material that was exposed to much lower electron dose. A similar argument holds for sample heating: small structures exposed to high dose are surrounded by bulks of material that can function as a heat sink, thereby reducing the effect of local sample heating. Those expectations however depend on the properties of the specimen, such as structural stability and thermal conduction and will need to be investigated on a case-to-case basis.

One concern with the method is that the edge detection algorithm can potentially miss small, isolated features. This happens with a relevant probability for features that have a size of approximately one pixel, are not attached to larger structures, or give a contrast to the background in the same order of magnitude as the noise of the initial scan. If a feature is missed by the edge detection algorithm, the remaining information about the structure is removed by the filter step and the feature will entirely disappear in the final image. While this behavior is acceptable for most applications where structure and morphology of larger features are of interest, it might be an issue for questions about small particles close to the resolution limit of the microscope. The proposed adaptive sampling method is not applicable in these cases.

In general, the mask generation procedure has a large potential for improvements. Promising approaches include more sophisticated edge detection algorithms, advanced image filters as preprocessing to the edge detection, morphological operations as post processing operations, and the inclusions of three-dimensional data from previous slides, in the case of FIB/SEM.

The proposed filter method depends on the assumption that the filter area consists of large, connected shapes. Preliminary experiments on more complex samples showed that if the HIA has a shape that cuts the filter area in many small, isolated shapes, the filter kernel becomes locally too small, which leads to visual artifacts. This effect can be eliminated by closing small holes in the HIA using morphological image processing operations. The filter method is also based on the assumption that no high frequency details are present in the filter area. If the sample has a complex and relevant texture even between sharp edges, as is the case in most biological applications, the proposed filter method is not applicable. A different reconstruction method based on exemplary-based inpainting algorithms is probably most promising in this case.

While the noise level in the HIA is determined by the pixel-dwell time of the second scan, the bandwidth characteristics in the filter area can be influenced by choosing an appropriate size of the filter kernel. But in order to avoid visual artifacts in the middle frequency range of about 5 pixels, the Gaussian kernel has to be chosen sufficiently large. In this case, structures of the intermediate frequency range are removed by the filter as well and the filter area receives a smooth appearance. Noise levels in the filter area are then much lower than in the HIA. Whether or not this poses a problem depends on the application. For most automated analysis algorithms, the different noise levels are unproblematic or even favorable. The human eye, however, is highly sensitive to perceive differences in the noise level of surfaces, registering every variation as artifact. In order to avoid these problems and generate visually pleasant images, it is probably a good idea to ensure that all parts of the final image have the same noise level. This can easily be achieved by mixing the correct portion of the initial noisy image into the final image as described in the methods section.

The method as presented in this paper is a proof-of-concept study. As the prototypical workflow involves switching software twice for each image acquired, the procedure is clearly not practical for FIB/SEM or STEM tomography with hundreds or thousands of slices. However, as all involved computations are straightforward, the method can in principle be integrated directly into microscope control packages such as FEI XTUi. Once this integration is performed, image based adaptive scanning can easily be selected in the software, thereby removing the technical restrictions of the prototypic workflow presented in this paper. In this case, the method has the potential to replace uniform sampling as the standard scanning mode, at least for a suitable class of sparse gradient specimens.

The basic idea described in this study is not restricted to FIB/SEM. In principal, the method can be extended to low dose scanning transmission electron microscopy (STEM) and STEM tomography as well. As with FIB/SEM, adaptive pixel-dwell times can help to reduce acquisition times and dose exposure for tilt series acquisition in STEM tomography. There are, however, a number of issues to be addressed when applying the concept to STEM. Thickness variations in the samples can create low frequency contrast that might unnecessarily trigger the edge detection. This issue can potentially be removed by means of a background removal using a rolling ball filter or a high pass filter as a preprocessing step to the edge detection. In high resolution STEM with a pixel size approaching atomic scale, lattice fringes and drift will raise concerns for the method. We therefore think that the method in the current form will be restricted to resolutions above the atomic scale. Another concern is the signal response speed. Current generation STEM systems use electromagnetic scanning, which has a much slower signal response compared to latest generation SEM that uses electrostatic scanning and can lead to a deformation of the PSF. This issue might, however, be resolved by future STEM systems that use electrostatic scanning. One last consideration for the application to STEM is, if the image processing methods presented in this study are useful at all in the case that a tilt series is recorded and the dataset is subject to tomography reconstruction prior to presentation. In this case, it is probably better to avoid both filtering and noise level adaptation and perform tomographic reconstruction from the raw data acquired from the microscope. Filtering operations can then be performed in three dimensions on the tomogram, if they are required. Other interesting areas of application of the ideas presented in this study are the combination of FIB/SEM with energy-dispersive X-ray spectroscopy (EDS). Provided a sufficiently responsive means to scan the specimen, the method could even be adapted to work with scanning X-ray microscopy with synchrotron radiation.

## Conclusions

Image based adaptive sampling was introduced as a new acquisition scheme for SEM. The method is based on the idea of scanning the specimen with adaptive pixel-dwell times. Areas containing high frequency details such as phase boundaries were scanned slower in order to generate a better SNR while homogeneous areas of low frequencies were scanned faster and the resulting noise was removed using a-posterior filtering. The critical step of this method was the automatic separation of the image in areas requiring a high dose and areas that can safely be filtered. As this analysis can only be performed effectively by considering the entire image, a two-step scanning process was applied. An initial image was acquired at low dose and the analysis was performed on this image. A second scan was then applied to only those regions of the image that require more dose. The final image was generated by filtering and combining both dataset. Compared to an image acquired with uniform sampling at the same total electron dose resulted in a factor of ≈ 3 lower quantitative pixel errors on average, and a factor of ≈ 3 better SNR, which implies that the electron dose and thus the acquisition time can be reduced by a factor of about ten. The methods can in principle be applied to two-dimensional low dose STEM and STEM tomography as well.

## Additional Information

**How to cite this article**: Dahmen, T. *et al.* Feature Adaptive Sampling for Scanning Electron Microscopy. *Sci. Rep.* **6**, 25350; doi: 10.1038/srep25350 (2016).

## Figures and Tables

**Table 1:** Pixel error between the reference scan (uniform sampling with 60 *μs* pixel-dwell time) and different scan modes with an average pixel-dwell time of 3 *μs* each.

Sampling method	Average pixel error	Maximum pixel error	SNR	PSNR	SSIM
adaptive	1.30% ± 1.35%	45.1%	25.7	32.0 dB	0.81
adaptive + noise adaptation	1.88% ± 1.65%	47.3%	18.6	32.0 dB	0.69
uniform	4.42% ± 3.46%	68.2%	8.3	25.0 dB	0.31
uniform + isotropic diffusion	1.58% ± 1.81%	55.2%	20.5	26.5 dB	0.78

All values are presented as percentage of the maximum pixel intensity of the reference scan for normalization. Additionally, signal-to-noise-ratio (SNR), peak signal-to-noise-ratio (PSNR) and structural similarity index (SSIM) are shown.

**Table 2:** Optimal settings for the edge detection algorithm depending on the pixel-dwell time.

pixel-dwell time	SNR	σ/pixels	A
100 *ns*	1.1	6	20%
300 *ns*	1.4	3	15%
1 *μs*	2.3	2.5	15%
3 *μs*	3.8	2	10%

σ is the standard deviation of the filter kernel for the edge detection algorithm, A is the size of the HIA normalized to the total size of the image.
